# An insilico analysis: three upregulated microRNAs as potential diagnostic biomarkers of Papillary Thyroid Carcinoma (PTC)

**DOI:** 10.1186/s43046-026-00350-1

**Published:** 2026-04-13

**Authors:** Maharani Putri Hermawan, Sari Eka Pratiwi, Ridha Ulfah, Eko Rustianto Suhardiman, Mahyarudin Mahyarudin

**Affiliations:** 1 Medical Study Programme, Faculty of Medicine, Tanjungpura University, Pontianak, Indonesia; 2 Department Biology and Pathobiology, Faculty of Medicine, Tanjungpura University, Pontianak, Indonesia; 3 Department of Community Medicine, Faculty of Medicine, Tanjungpura University, Pontianak, Indonesia; 4 Department of Surgical Oncology, Soedarso General Hospital, Pontianak, Indonesia; 5 Department of Microbiology, Faculty of Medicine, Tanjungpura University, Pontianak, Indonesia

**Keywords:** MicroRNA, Papillary thyroid carcinoma, In silico, Diagnosis, Biomarkers

## Abstract

**Background:**

Papillary thyroid carcinoma (PTC) represents the most prevalent subtype of thyroid cancer. Accumulating evidence indicates that specific microRNAs are consistently dysregulated in PTC and may hold value as biomarker candidates. Accordingly, this study aims to prioritize consistently upregulated microRNAs associated with PTC using an integrative in silico analysis of publicly available datasets.

**Methods:**

This study conducted a comprehensive analysis of miRNA expression patterns using datasets available through A Database of Differentially Expressed miRNAs in Human Cancers (dbDEMC). The dataset was then processed through a data mining approach with a cutoff of P-value < 0.05 and log_2_FC > 1.5 to identify miRNAs that were significantly and consistently upregulated in the datasets. The target genes are predicted through miRDIP, miRTarBase, miRPathDB, and GEPIA2. Gene ontology and pathway enrichment analysis were performed in ShinyGO and EnrichR. To assess the diagnostic ability of the three miRNAs, CancerMIRNome is used to identify the ROC curve analysis results of each miRNA.

**Results:**

This exploratory in silico analysis identified 85 differentially expressed miRNAs in PTC, with hsa-miR-221-3p, hsa-miR-222-3p, and hsa-miR-146b-5p consistently upregulated across datasets. Functional enrichment and TCGA-based ROC analyses suggest that these miRNAs may have biological and discriminatory relevance in PTC. However, these findings are hypothesis-generating and require further experimental and clinical validation before any diagnostic application can be considered.

**Conclusion:**

In summary, our study identified the potential of hsa-miR-221-3p, hsa-miR-222-3p, and hsa-miR-146b-5p as potential diagnostic biomarkers of PTC.

**Supplementary Information:**

The online version contains supplementary material available at 10.1186/s43046-026-00350-1.

## Background

Thyroid cancer is the most common malignancy in the endocrine system [1]. The incidence of thyroid cancer has increased significantly between 2018 and 2022. Globally, there was an increase by 1,4 times, while in Indonesia alone the incidence of thyroid cancer increased by 3,7 times [2, 3]. Thyroid cancer is classified into four major subtypes: papillary, follicular, medullary, and anaplastic. Of the four types, the papillary subtype, which will be referred to as papillary thyroid carcinoma (PTC), is the variant of thyroid cancer with the highest incidence, accounting for 80–85% of all thyroid cancer cases. Although PTC is considered a low-mortality cancer with a 5-year survival rate of 80–95%, metastasis in PTC is very common. The metastasis of PTC is 30–40% to regional lymph nodes in the neck area, but it may reach distant organs such as the lungs and the bone [1].

In general, the diagnosis of PTC is established through several supporting examinations, such as ultrasonography, thyroid function, and Fine Needle Aspiration Biopsy (FNAB) [1] The gold standard in the diagnosis of thyroid cancer is the FNAB procedure [4] FNAB is often combined with ultrasonography to improve the accuracy of the results. However, according to the American Thyroid Association (ATA) guidelines, the FNAB procedure is recommended to be performed only on nodules >1 cm in size [5] The requirement of performing FNAB is a hurdle in detecting PTCs that are evolving into small-sized carcinomas over the years, even less than 1 cm [6]. In addition, the cytology results through the FNAB procedure do not always indicate malignancy, and other tests, such as molecular testing, are needed to determine the appropriate course of action in treating thyroid nodules [7].

During this phase, microRNA (miRNA) can play a role as one of the supports for the diagnosis of PTC. MicroRNA is a type of non-coding RNA and plays a role as a post-transcriptional regulator that can suppress the process of gene expression by degrading the messenger RNA (mRNA) involved [7]. In cancer tissue, miRNA can be categorized as oncomiR, which supports the process of cancer cell formation and oncosuppressormiR, which suppressed the cancer cell formation. By analyzing the differences in miRNA expression between PTC tissues and normal tissues, several miRNAs will be identified that have the potential to become diagnostic biomarker candidates for PTC [8] In addition, miRNAs have high stability and specificity and can be found in tissues and body fluids such as blood, urine, and other body fluids, thus having great potential to become a minimally to non-invasive diagnostic tool [9].

Consistent differentially expressed microRNA in PTC can be identified with the in-silico method, where researchers are able to reinvestigate previous studies and with the help of bioinformatics technology. In silico methods can be used as a preliminary study in identifying biomarker candidates without having to incur a great expense or require a lengthy duration of time [10]. This study employed a cross-dataset consistency approach to prioritize genes that show reproducible signals across multiple datasets as potential biomarker candidates for PTC, rather than aiming to identify novel markers. Therefore, this study aims to identify prioritized upregulated microRNAs that may serve as potential diagnostic biomarkers of PTC with an in silico approach.

## Methods

### Data collection

Data collection begins with retrieving datasets through Database of Differentially Expressed miRNAs in Human Cancers (dbDEMC) [11] by choosing “thyroid cancer”, “cancer vs. normal”, “Homo sapien”, and “microarray”. The data that appears will be sorted again, leaving only the dataset with GEO source IDs, as the data will be reprocessed through GEO2R [12]. Differential expression analysis was conducted using GEO2R, a web-based interface implementing the limma package. GEO2R operates on normalized expression matrices provided by the original data submitters, and no additional normalization was applied. Each GEO dataset was analyzed independently to identify differentially expressed miRNAs. Batch effect correction across datasets was not performed due to differences in microarray platforms; therefore, comparisons across datasets were based on the consistency of expression direction rather than absolute log2fc values. Then, the obtained data will be processed to be grouped according to the type of sample, namely, normal and cancer.

### Identification of differentially expressed MiRNAs (DEMs)

Datasets that have been downloaded through GEO2R will be visualized as a volcano plot through VolcaNoseR [13] with a cut off of P-value < 0.05 and |log2FC| >1.5. In addition to forming a volcano plot, the GEO2R data will be reprocessed using the Orange app [14] to obtain miRNAs that are upregulated in cancer samples with a cut-off of P-value < 0.05 and |log2FC| >1.5. MiRNA candidates were then selected based on log2FC value, significance, and number of occurrences in the three datasets that are used in this study. Multiple testing correction was not applied during the initial miRNA screening, as the analysis was designed to be exploratory and hypothesis-generating rather than confirmatory. To mitigate false positives, candidate miRNAs were further filtered based on consistency of expression direction across independent datasets and subsequent validation using TCGA-derived ROC analysis.

### Identification of differentially regulated genes (DEGs)

Once the candidate miRNAs were found, the predicted target genes of each miRNA candidates were identified through miRDIP [15], miRTarBase [16], and miRPathDB [17]. In addition, GEPIA2 [18] was used to find deregulated genes in thyroid cancer using LIMMA, with |log2FC| > 1.5 and a p-value of 0.05. To identify differentially expressed genes (DEGs), we integrated the predicted target genes of the miRNA candidates with genes reported to be deregulated in PTC. The genes that appeared in all databases were selected as target genes. The selection of target genes was carried out by creating a Venn diagram based on acquired dataset using the Bioinformatics and Evolutionary Genomics web tool. The intersection identified by the Venn diagram was considered the set of target genes selected for further analysis. Signaling pathways of Kyoto Encyclopedia of Genes and Genomes (KEGG) and Gene Ontology (GO) of the target genes were analyzed through EnrichR [19] and ShinyGo [20].

## Results

### Differentially expressed MiRNAs (DEMs) datasets

**Fig. 1:**
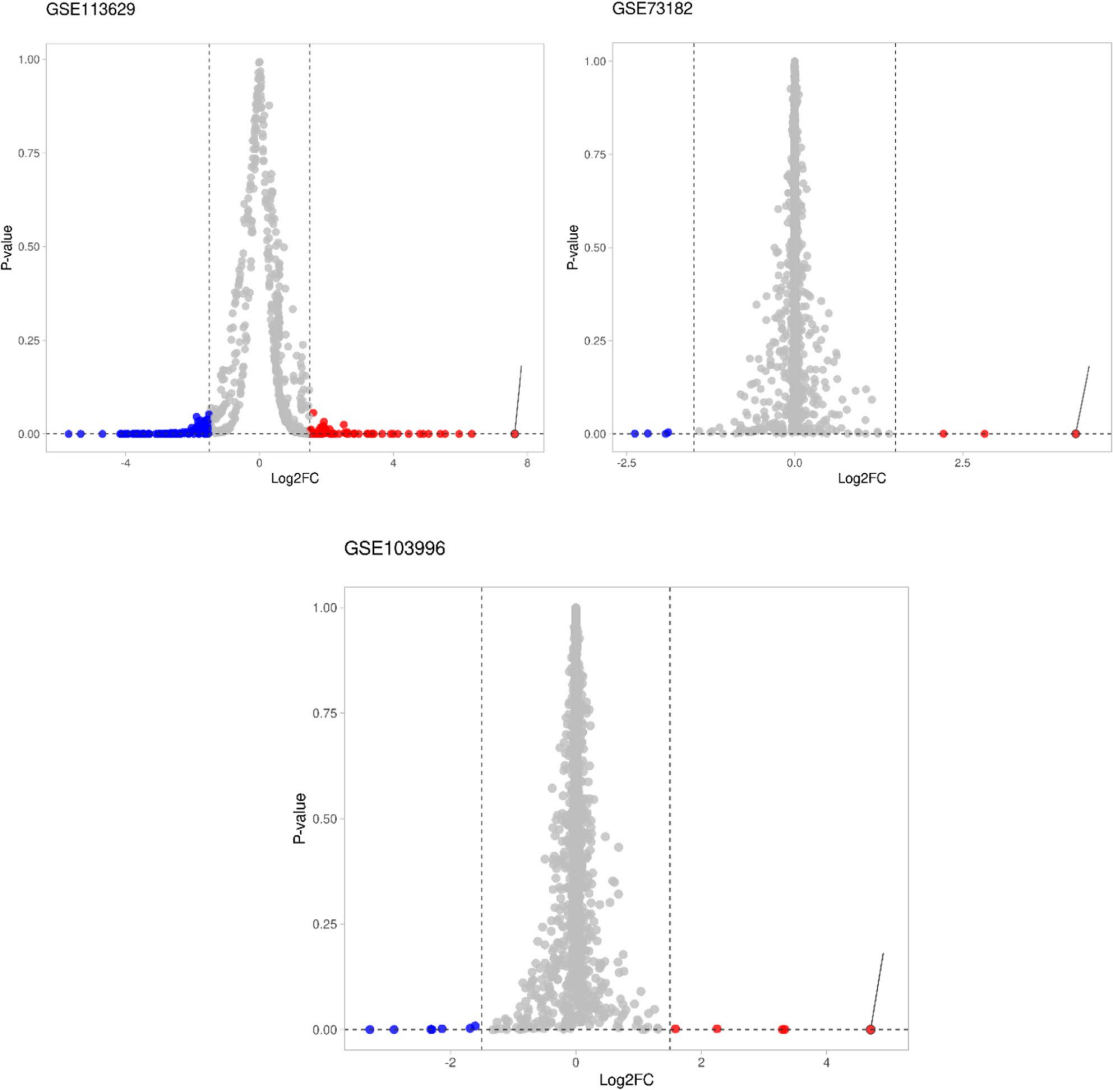
Eligible datasets volcano plot. Red indicates miRNAs with increased expression, blue indicates decreased expression, and grey indicates insignificant changes

In this research, we found and utilized three datasets acquired from dbDEMC that fulfill our criteria of an eligible dataset, which are GSE113629, GSE73182, and GSE103996. These datasets contained deregulated miRNAs derived exclusively from tissue samples, as blood-based datasets did not include GEO accession IDs. Consequently, they did not meet the predefined inclusion criteria and were excluded from the analysis. To visually identify the differentially expressed miRNAs in each dataset, volcano plots are made through VolcanoNoseR (Fig. 1).

### Distinguishing the miRNAs candidate

The eligible datasets acquired from dbDEMC are analyzed through the Orange app to obtain the significantly upregulated miRNA with a cut-off value of P-value < 0.05 and Log_2_FC > 1.5. The data analyzed through the Orange app will then be processed in Microsoft Excel to calculate the average Log_2_FC and P-value, also to determine the number of miRNA occurrences using the Pivot Table feature. A total of 85 upregulated miRNAs were then identified (Supplementary Table 1). Based on the Log_2_FC value, significance (P-value < 0.05), and number of occurrences in the three datasets, hsa-miR-221-3p, hsa-miR-222-3p, and hsa-miR-146b-5 are selected for further analysis (Table 1). These three miRNAs have the highest Log2FC average, which is also statistically relevant and can be found consistently in all three datasets used and the CancermiRnome TCGA datasets compared to the other 82 miRNAs that we didn’t analyze further, thus why they are chosen to be our miRNA candidates.

**Table 1:** Top three upregulated miRNAs

MiRNA	Average Log2FC	Average *P*-value	CancermiRnome *P*-value&AUC score	Occurence
hsa-miR-146b-5p	5.50	0.00003	***&0.91	GSE73182 GSE103996 GSE113629
hsa-miR-221-3p	3.72	0.000044	***&0.93	GSE73182 GSE103996 GSE113629
hsa-miR-222-3p	3.44	0.0001	***&0.93	GSE73182 GSE103996 GSE113629

(*) represent t-test results, ***=*P*-value < 0.0001

### Target genes discovery

**Fig. 2:**
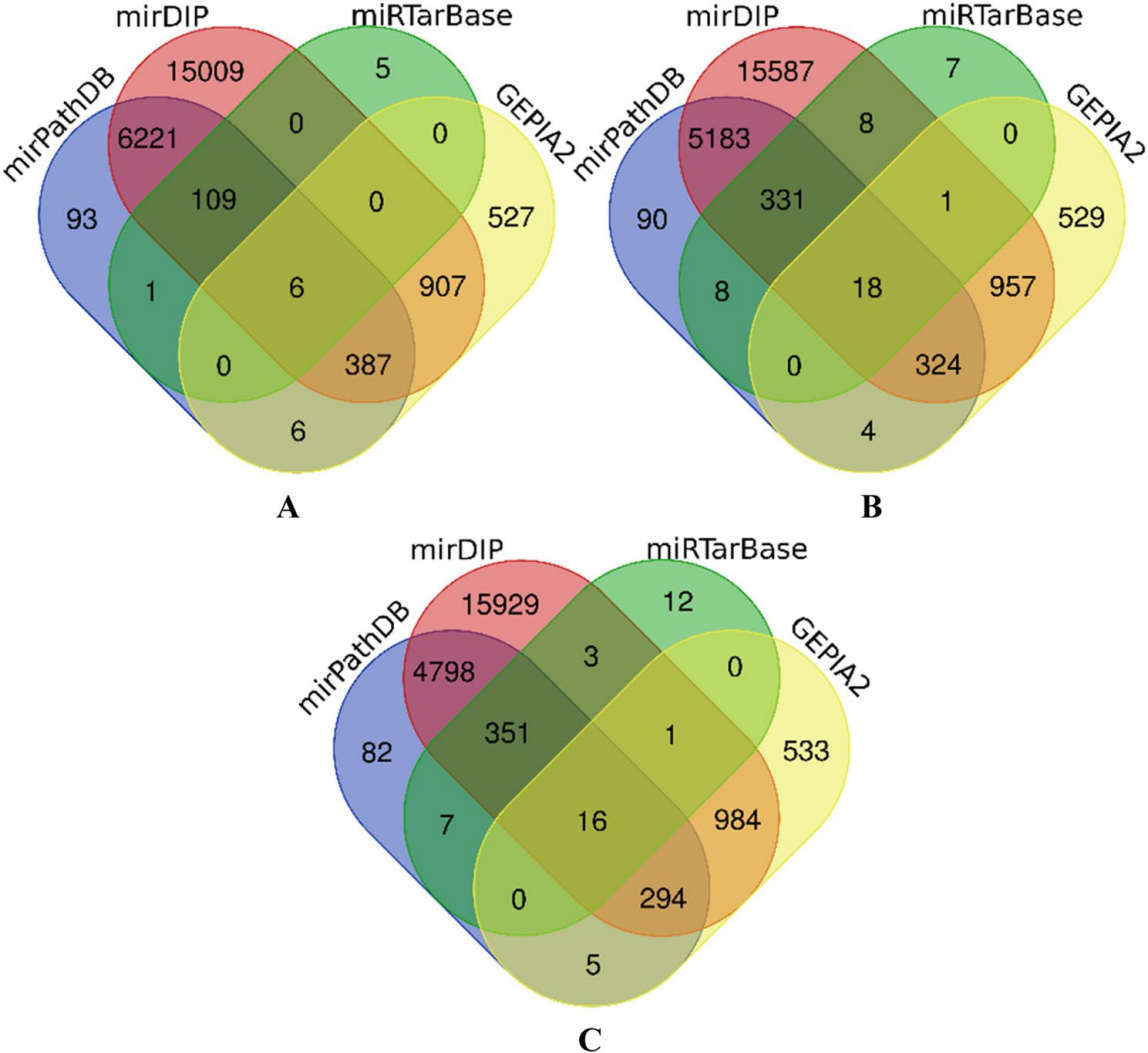
miRNAs candidates target genes venn diagram (**A**) Hsa-miR0146b-5p, (**B**) Hsa-miR-221-3p, (**C**) Hsa-miR-222-3p

The target genes of our miRNA candidates are chosen if they appeared in all of the databases that we used to obtain differentially regulated genes in thyroid cancer and the potentially targeted genes of our miRNA candidates. Through Bioinformatics and Evolutionary Genomics web tool, we made a Venn diagram to filter the eligible target genes (Fig. 2). The list of target genes for each miRNA candidate is provided in Supplementary Table 2.

### Significance and differentiating performance of miRNA candidates

**Fig. 3:**
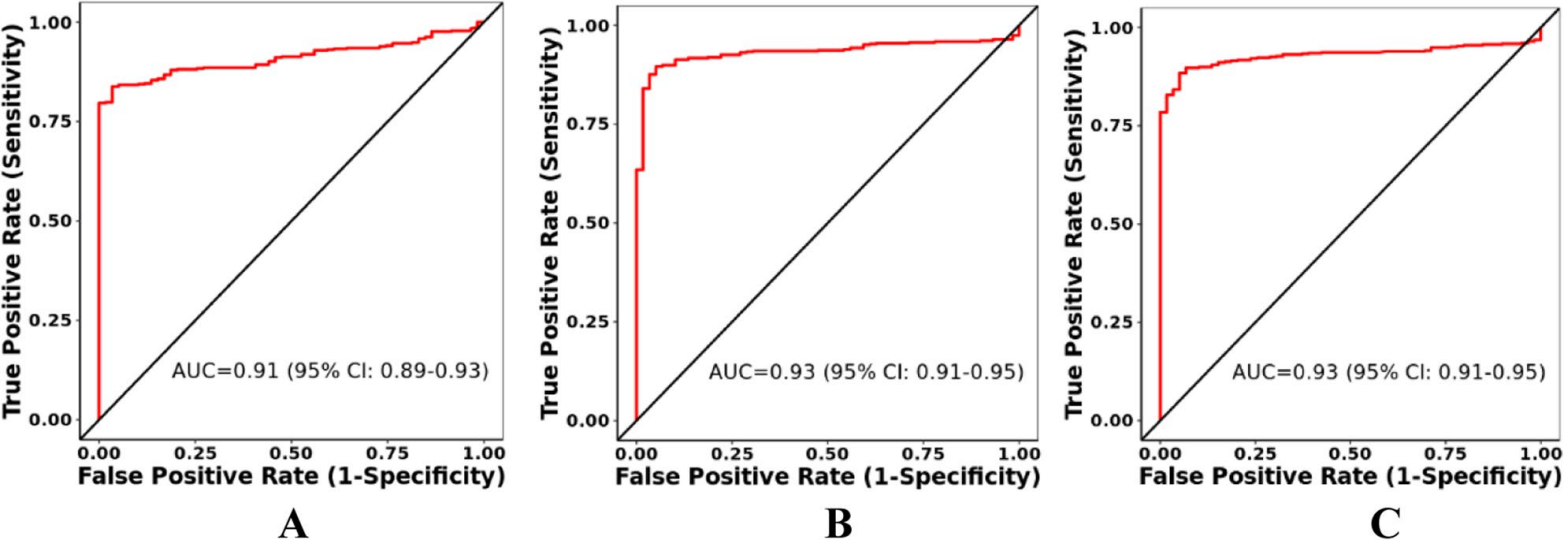
ROC curve of (**A**) Hsa-miR0146b-5p, (**B**) Hsa-miR-221-3p, (**C**) Hsa-miR-222-3p

**Fig. 4:**
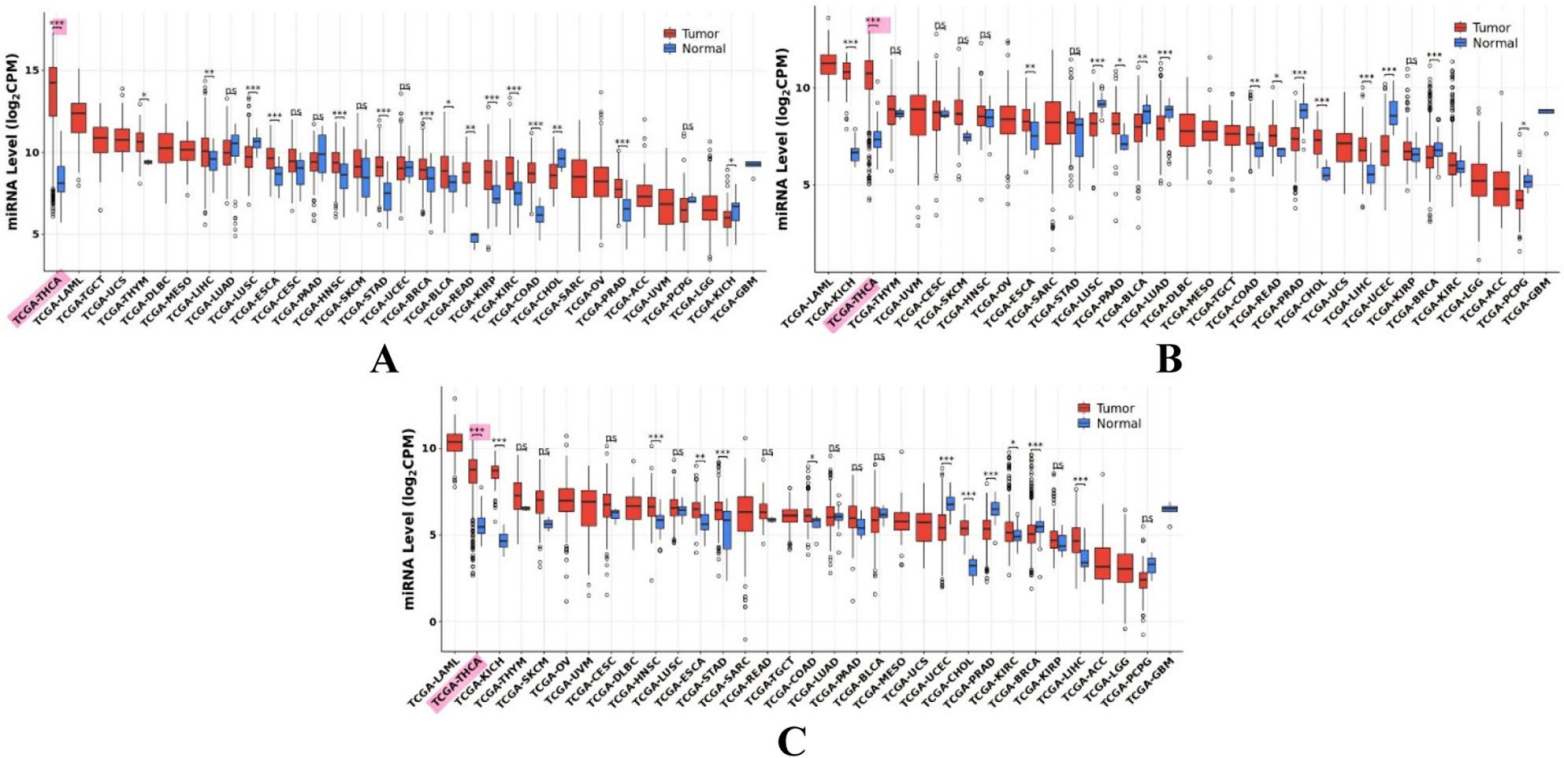
Significance of (**A**) Hsa-miR0146b-5p, (**B**) Hsa-miR-221-3p, (**C**) Hsa-miR-222-3p on various cancers. Thyroid cancer is abbreviated as THCA (highlighted in pink). (*) represent t-test results;*=*P*-valure<0.01, **=*P*-value<0.001, ***=*P*-value<0.0001, ns = not significant

In order to assess the discriminatory values and the significance of the selected miRNAs between thyroid cancer tissues and matched normal tissue, CancermiRNome is used to identify the corresponding AUC score of each miRNA and its expression significance on the Cancer Genome Atlas (TCGA) tumor and normal samples. Hsa-miR-221-3p, hsa-miR-222-3p, and hsa-miR-146b-5p exhibit good discriminatory ability with the AUC score > 0,9 (Fig. 3). The ROC analysis demonstrated moderate to high discriminatory ability in the analyzed dataset, as reflected by the AUC values. However, confidence intervals and comparative statistical testing were not performed; therefore, the diagnostic performance should be interpreted with caution. Whereas for their significance in thyroid cancer, a p-value < 0,0001 was obtained for each of the miRNAs as shown in Fig. 4. This finding shows that each of the miRNA are significantly upregulated in thyroid cancer.

### Target genes prediction and functional enrichment analysis

**Fig. 5:**
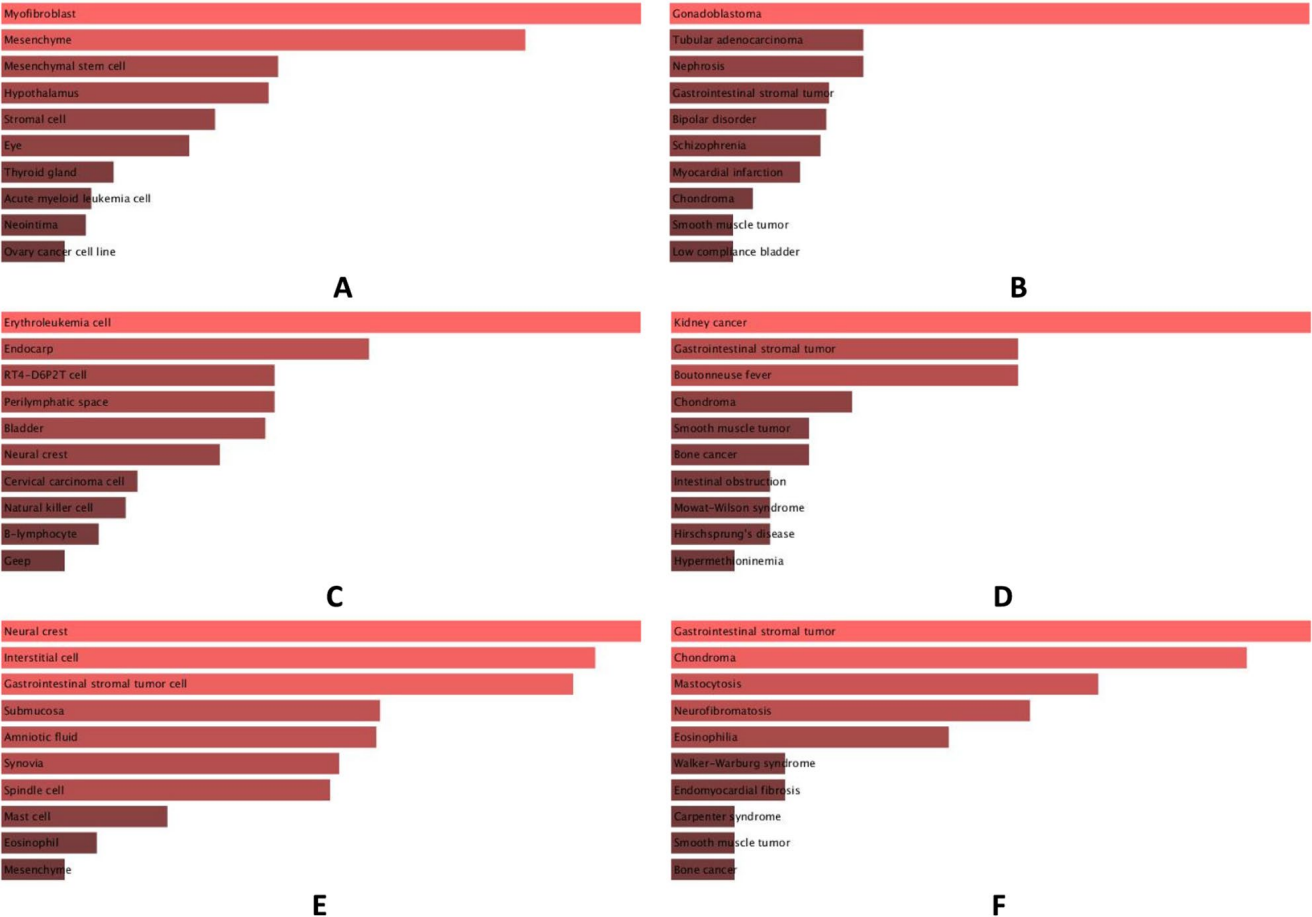
Top 10 tissues and diseases of the target genes of (**A**, **B**) Hsa-miR-221-3p, (**C**, **D**) Hsa-miR-222-3p, (**E**, **F**) Hsa-miR-146b-5p. The color and the length indicate the significance of the term; the longer and the lighter the bar is, the more significant the term is

**Fig. 6:**
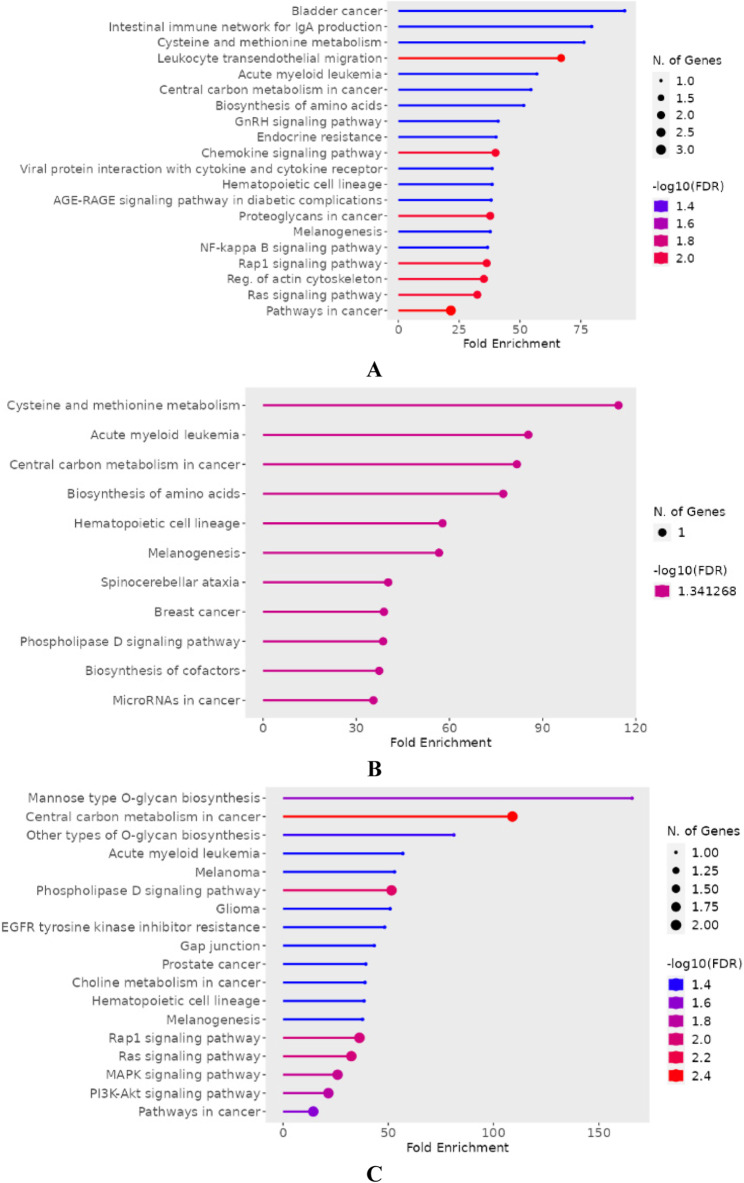
Signaling pathways diagram of (**A**) Hsa-miR-221-3p, (**B**) Hsa-miR-222-3p, and (**C**) Hsa-miR-146b-5p target genes. The size of the circle represents the number of genes involved, the length of the line represents the fold enrichment value, and the color difference represents the False Discovery Rate (FDR) value

The overlapping target genes of each miRNA that were discovered are used to perform enrichment analysis. Several enriched KEGG pathways were driven by a limited number of genes, which may reflect exploratory associations rather than robust pathway-level activation. Hsa-miR-221-3p target genes are involved in phospholipase activity, cell migration, differentiation, chemotaxis, and epithelial cell migration-related biological processes (S1). The thyroid was also one of the top 10 tissues that involved hsa-miR-221-3p target genes (Fig. 5(A)). The enrichment KEGG pathways were mainly associated with pathways in cancers, involving 3 genes and a -log10 2.0 false discovery rate (FDR) (Fig. 6(A)). Hsa-miR-222-3p target genes are involved in epithelial cell migration, DNA replication, actin cytoskeleton reorganization, etc. They were also found in carcinoma disease with a *p-value* of 0,037185, but were not included in the top 10 diseases and were not significantly expressed in the thyroid gland (Fig. 5 (D)). The enrichment of KEGG pathways was mainly associated with central carbon metabolism in cancer, involving a single gene (Fig. 6(B)). Hsa-miR-146b-5 target genes were not significantly found in thyroid gland tissue and carcinoma diseases. But they are involved in epithelial cell migration, MAPK cascade positive regulator, and stem cell differentiation-related biological processes. The enrichment KEGG pathways were mainly associated with central carbon metabolism in cancer, involving 2 genes, a 0,0038 FDR, and a fold enrichment score of 109 (Fig. 6(C)). These pathways may represent potential biological processes associated with the identified targets, although the limited gene representation warrants cautious interpretation.

## Discussion

MicroRNAs are small pieces of RNA with a size of 21–23 nucleotides that are not translated into proteins and function as regulators of gene expression. During the transcription process, microRNA (miRNA) attaches to the 3′ untranslated region (3′ UTR) of messenger RNA (mRNA). This mechanism will cause the mRNA to be degraded and suppress the gene transcription process [7]. Various studies have proven that miRNAs have a major involvement in the development of cancer cells, especially in the focus of this study, namely Papillary Thyroid Carcinoma (PTC). Research conducted by Celakovsky et al. found that increased expression of miR-146b was specifically found in PTC patients associated with BRAF gene mutations, and increased expression of miR-221 in recurrent PTC patients [21].

This study reported that hsa-miR-221-3p, hsa-miR-222-3p, and hsa-miR-146b-p are the top three significantly upregulated miRNAs in dbDEMC’s PTC sample using an in silico approach. A previous in silico study unveiled a three-microRNA signature (miR-21, miR-584, and miR-155) as a diagnostic marker in clear cell renal cancer (KIRC) using univariate Cox regression analysis [22]. In the present study, the eligible dataset acquired from dbDEMC was analyzed using the Orange app with a cut-off of P-value < 0.05 and log2FC > 1.5 to identify upregulated miRNAs. These upregulated miRNAs were then sorted based on their occurrence in the used datasets, average P-value, and log_2_FC value. We found that hsa-miR-221-3p, hsa-miR-222-3p, and hsa-miR-146b-p matched our criteria of a significantly upregulated miRNA as they appear in all three datasets along with a high average log_2_FC value and proved to be statistically significant (P-value < 0.05). These top three upregulated miRNAs were then confirmed to be significantly upregulated in TCGA samples that are stored in the CancermiRNome repository. Our findings also show that each of our top three upregulated microRNAs has an excellent ability in distinguishing PTC from normal tissue, with the area under the curve (AUC) score above 0.9 although further experimental research are needed to prove it’s clinical usability as a diagnostic biomarker [23].

Our findings are consistent with previous studies that stated these three miRNAs are also significantly upregulated in PTC. In vivo and in vitro studies by Diao et al. suggest that miR-221 promotes the proliferation and invasion of PTC cells by targeting the TIMP3 gene, a tumor suppressor gene [24]. Jahanbani et al. stated that hsa-mir-222-3p has an increased expression in tissue and blood samples of PTC patients [25]. Jia et al. revealed that hsa-miR-146b-5p has been proven in vivo and in vitro to be upregulated and act as an oncomiR in PTC carcinogenesis by targeting the CCDC6 gene that codes a mutated chimeric protein of the RET proto-oncogene [26]. Emerging evidence suggests that miRNA expression profiles may complement standard diagnostic tools in PTC. For example, miR-146b and miR-181b demonstrated diagnostic discrimination between benign and malignant thyroid nodules in FNAB specimens, which could be particularly useful for indeterminate cytology cases where traditional FNAB has limitations (e.g., sensitivity/specificity) and current practice often includes molecular testing such as BRAF mutation analysis to improve accuracy [27]. Meta-analyses of circulating miRNAs, including miR-146 and miR-222, report overall good diagnostic performance (summary AUROC ~ 0.89) for differentiating PTC from benign nodules and controls, aligning with but not yet exceeding the diagnostic benchmarks established by FNAB and molecular panels, suggesting that miRNAs could provide incremental value when combined with existing methods rather than replace them [28]. In line with these findings, another meta-analysis reported that miR-146 exhibited the highest sensitivity (80.88%) for distinguishing PTC from benign thyroid nodules, whereas miR-222 demonstrated the highest specificity (80.7%), further supporting the potential complementary diagnostic roles of individual miRNAs rather than reliance on a single marker [29]. Additionally, the observed association between miRNA upregulation, especially miR-146b, and BRAF mutation status in PTC tissue suggests a potential role for miRNAs in integrated molecular diagnostic strategies, although the clinical utility of such integration requires further prospective validation [21]. Finally, the dynamic changes in circulating miRNA levels pre- and post-thyroidectomy highlight the possibility of miRNAs serving as non-invasive biomarkers for disease status monitoring, adding a dimension not captured by static cytological or genetic tests alone [30].

As mentioned before, miRNAs’ role in cancer is either to support or suppress oncogenesis. They indirectly affect certain pathways that could lead to cancer cell formation by restraining several gene expressions, mostly the tumor suppressor genes. Systematic reviews have documented the oncogenic roles of miR-221/222 in a wide range of solid tumors, where increased expression promotes proliferation, invasion, and resistance through modulation of key oncogenic pathways such as PI3K/AKT and EMT-related signaling, and by targeting tumor suppressors like PTEN and cell cycle regulators (e.g., p27, p57) in cancers including breast, colorectal, and hepatic carcinoma [31–33]. Similarly, meta-analytic evidence supports the association of elevated miR-146b and miR-221/222 with aggressive disease and recurrence in thyroid cancer, suggesting that these miRNAs are linked to malignant phenotypes rather than being unique markers of PTC alone [29, 34]. The broad involvement of these miRNAs across diverse tumor contexts suggests they may reflect shared oncogenic stress responses and common dysregulated networks such as MAPK/ERK and PI3K/AKT signaling, rather than exclusively PTC-specific molecular changes. Within this framework, the value of these molecules may lie in their robust reproducibility across datasets and their potential to prioritize biologically relevant miRNAs for future validation, while acknowledging that further mechanistic and clinical studies are needed to delineate their specificity and diagnostic utility in PTC.

To further explore the potential biological relevance of the identified miRNAs, predicted target genes were compiled and subjected to functional enrichment analysis. The results suggest that hsa-miR-221-3p may regulate a set of genes, including MMP2, KIT, and CXCL12, which have been previously associated with angiogenesis-related processes in cancer (Supplementary Fig. 1). Likewise, enrichment analysis of predicted targets of hsa-miR-146b-5p, particularly KIT and PDGFRA, indicates a possible association with cancer-related metabolic pathways and ERK/MAPK signaling (Supplementary Fig. 2). In addition, hsa-miR-222-3p was predicted to target KIT. Collectively, these in silico predictions indicate that KIT may represent a shared putative target of the three miRNAs, warranting further investigation in experimental settings. KIT or KIT proto-oncogene encodes a mitogen-activated receptor tyrosine kinase (RTK). These RTKs are the activators of the ERK/MAPK pathway. Activation of this pathway plays an essential role in cell proliferation, angiogenesis, and invasion [35]. In PTC, several studies have shown that KIT gene expression is decreased when compared to benign samples [36–38]. On the contrary, overexpression of KIT will decrease the malignant features of thyroid cancer cells and the proliferation ability of tumor cells, suggesting that this gene is involved in the differentiation of normal cells into cancer [36, 38]. An experimental study combined with bioinformatics analysis also stated that his gene is also found to inhibit the immune escape ability of thyroid cancer cells by blocking the activation of the MAPK pathway and downregulating Programmed death-ligand 1 (PD-L1) an immunosuppresant molecule [37]. Current evidences suggest that KIT may function as a tumor-suppressive regulator in PTC; however, this role remains context-dependent and requires further mechanistic validation.

Taken together, these observations raise the possibility that dysregulation of KIT-related pathways may be relevant to PTC biology. However, it is important to emphasize that the present findings are based solely on in silico target prediction and pathway enrichment analyses. The proposed involvement of KIT as a common regulatory node should therefore be regarded as hypothesis-generating rather than mechanistically established. Further studies incorporating expression correlation analyses, functional assays, and experimental validation are required to clarify whether and how the identified miRNAs may influence KIT regulation and contribute to PTC oncogenesis.

## Conclusion

In conclusion, through an integrative in silico analysis of differentially expressed miRNAs across multiple independent datasets, this study highlights hsa-miR-221-3p, hsa-miR-222-3p, and hsa-miR-146b as prioritized miRNA candidates associated with papillary thyroid carcinoma. These miRNAs consistently exhibited altered expression patterns and demonstrated discriminatory potential between PTC and normal samples in TCGA-based ROC analyses.

In addition, our findings suggest a possible regulatory association between the identified miRNAs and the downregulated KIT gene, a molecule known to be involved in ERK/MAPK-related cellular processes. While this observation may offer insight into PTC-related molecular interactions, it should be regarded as hypothesis-generating rather than mechanistically established. Importantly, this study is limited by its exploratory in silico design and the absence of experimental or prospective clinical validation. Therefore, the identified miRNAs should not be interpreted as ready-to-use diagnostic biomarkers. Instead, they represent promising candidates that warrant further validation through functional experiments and well-designed clinical studies before any diagnostic application can be considered.

## Supplementary Information

Supplementary Material 1.

Supplementary Material 2.

## Data Availability

1. DEMs are acquired through dbDEMC database under experiment number EXP00548 and are available at the following URL: [https://www.biosino.org/dbDEMC/experiment/detail/EXP00548]. 2. DEMs data are acquired through dbDEMC database under experiment number EXP00585 and are available at the following URL: [https://www.biosino.org/dbDEMC/experiment/detail/EXP00585]. 3. DEMs data are acquired through dbDEMC database under experiment number EXP00452 and are available at the following URL: [https://www.biosino.org/dbDEMC/experiment/detail/EXP00452]. 4. DEMs microarray data were deposited into Gene Expression Omnimbus database under accession number GSE113629 and are available at the following URL: [https://www.ncbi.nlm.nih.gov/geo/query/acc.cgi?acc=GSE113629]. 5. DEMs microarray data were deposited into Gene Expression Omnimbus database under accession number GSE103996 and are available at the following URL: [https://www.ncbi.nlm.nih.gov/geo/query/acc.cgi?acc=GSE103996]. 6. DEMs microarray data were deposited into Gene Expression Omnimbus database under accession number GSE73182 and are available at the following URL: [https://www.ncbi.nlm.nih.gov/geo/query/acc.cgi?acc=GSE73182]. 7. Target genes datasets are acquired through mirDIP: [https://ophid.utoronto.ca/mirDIP/]., miRPathDB: [https://mpd.bioinf.uni-sb.de/]., miRTarBase: [https://mirtarbase.cuhk.edu.cn/~miRTarBase/miRTarBase_2025/php/index.php]., and GEPIA2 : [https://gepia.cancer-pku.cn/]. 8. Target genes Venn diagram is made through Bioinformatics & Evolutionary Genomics web tool and are available at the following URL: [https://bioinformatics.psb.ugent.be/webtools/Venn/]. 9. Functional enrichment analysis is done through ShinyGO: [https://bioinformatics.sdstate.edu/go/]. and Enrichr repository: [https://maayanlab.cloud/Enrichr/].

## Abbreviations

PTC: Papillary Thyroid Carcinoma
miRNA: MicroRNA
DEMs: Differentially Expressed MiRNAs
DEGs: Differentially Expressed Genes
FNAB: Fine Needle Aspiration Biopsy
ROC: Receiver Operating Characteristic Curve
AUC: Area Under the Curve
MAPK: Mitogen-Activated Pathway Kinase
PDGFRA: Platelet-Derived Growth Factor Receptor Alpha
MMP2: Matrix Metalloproteinase 2
CXCL12: C-X-C Motif Chemokine Ligand 12
