# Supplementary material for: An insilico analysis: three upregulated microRNAs as potential diagnostic biomarkers of Papillary Thyroid Carcinoma (PTC)
Source: J Egypt Natl Canc Inst. 2026 Apr 13;38:11. doi: 10.1186/s43046-026-00350-1 (PMC13313286; doi:10.1186/s43046-026-00350-1)
Supplement: Supplementary file 1 — Supplementary Material 1. [file 43046_2026_350_MOESM1_ESM.docx]

**Suppelementary data**

**
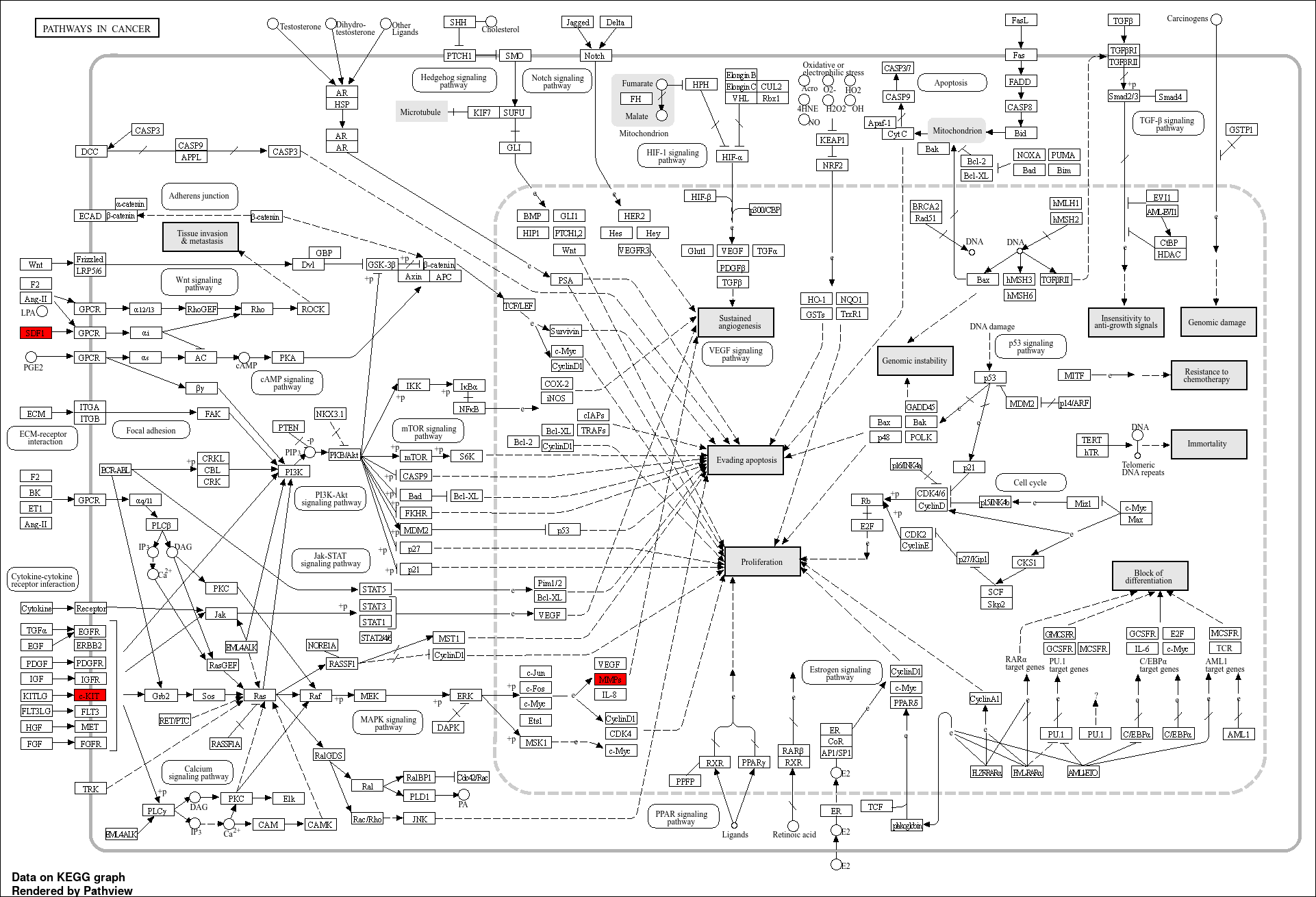
**

**Supplementary figure 1** KEGG Pathway analysis of hsa-miR-221-3p

**
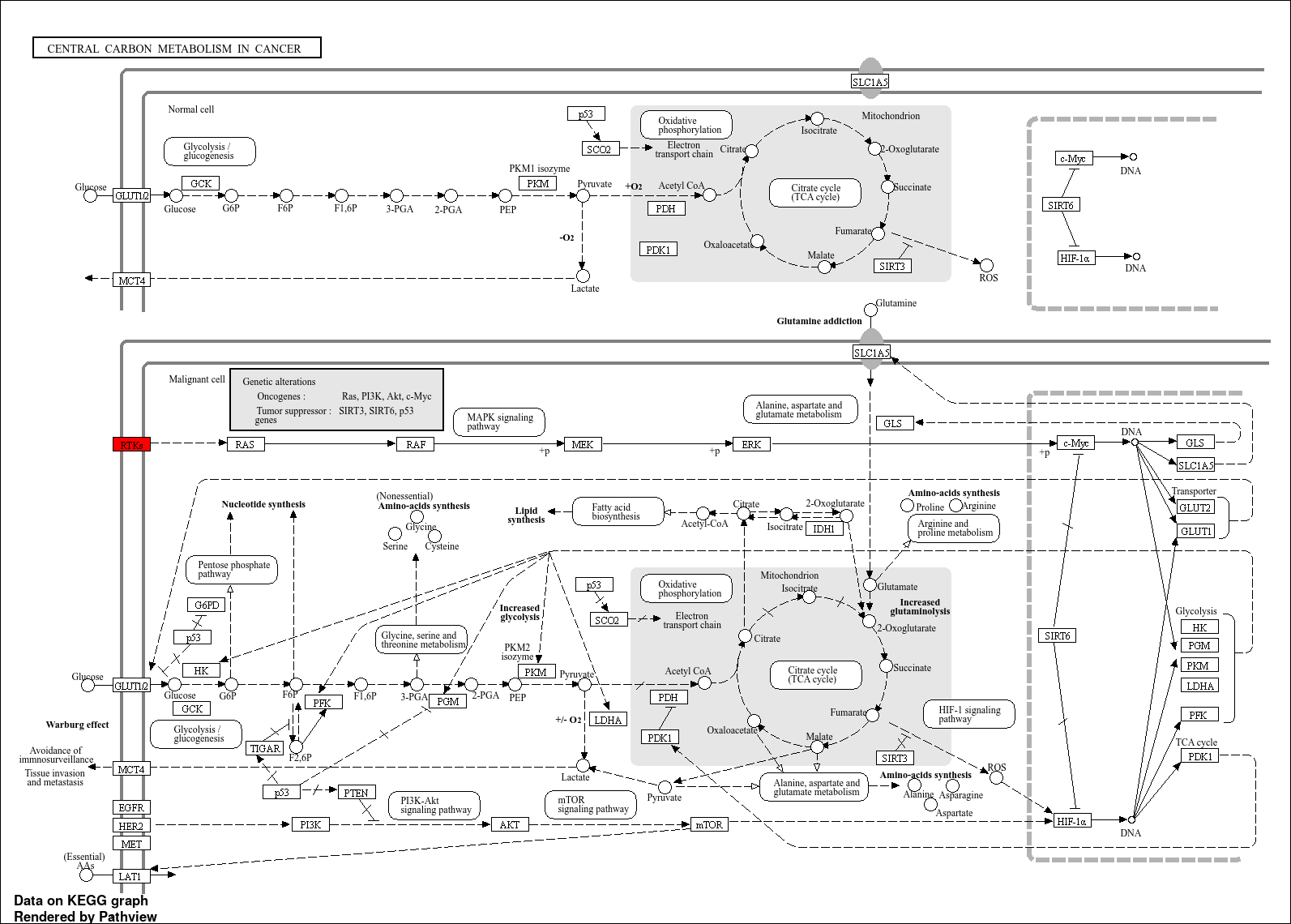
**

**B**

**Supplementary figure 2** KEGG Pathway analysis of hsa-miR-222-3p and hsa-miR-146b-5p target genes.
