# Supplementary material for: An insilico analysis: three upregulated microRNAs as potential diagnostic biomarkers of Papillary Thyroid Carcinoma (PTC)
Source: J Egypt Natl Canc Inst. 2026 Apr 13;38:11. doi: 10.1186/s43046-026-00350-1 (PMC13313286; doi:10.1186/s43046-026-00350-1)
Supplement: Supplementary file 2 — Supplementary Material 2. [file 43046_2026_350_MOESM2_ESM.docx]

**Supplementary Table 1** Upregulated miRNAs in Papillary Thyroid Carcinoma. Texts highlighted in red are the selected miRNAs for further analysis.

| **miRNA** | **Count of dataset occurrence** | **Average of LOG2FC** | **Average of P-VALUE** |
| --- | --- | --- | --- |
| hsa-miR-222-3p | 3 | 3.446133333 | 0.000105557 |
| hsa-miR-221-3p | 3 | 3.722763333 | 4.485E-05 |
| hsa-miR-146b-5p | 3 | 5.501663333 | 3.05068E-05 |
| hsa-miR-221-5p | 2 | 3.2385 | 0.000875 |
| hsa-miR-551b-3p | 2 | 4.296135 | 0.000985068 |
| hsa-miR-7-1-3p | 1 | 1.53093 | 0.00241 |
| hsa-miR-4701-5p | 1 | 3.21258 | 0.000519 |
| hsa-miR-3654 | 1 | 1.99209 | 0.00217 |
| hsa-miR-132-5p | 1 | 2.02525 | 0.00237 |
| hsa-miR-625-5p | 1 | 1.51489 | 0.00833 |
| hsa-miR-135a-5p | 1 | 3.33666 | 0.0000347 |
| hsa-miR-34a-3p | 1 | 4.13049 | 0.00000182 |
| hsa-miR-135b-5p | 1 | 2.16233 | 0.0000124 |
| hsa-miR-424-5p | 1 | 2.17028 | 0.0000658 |
| hsa-miR-141-5p | 1 | 2.58934 | 0.000858 |
| hsa-miR-489-3p | 1 | 1.92496 | 0.00183 |
| hsa-miR-146b-3p | 1 | 5.54076 | 2.02E-10 |
| hsa-miR-6858-3p | 1 | 1.92496 | 0.00809 |
| hsa-let-7i-5p | 1 | 1.82437 | 0.0000893 |
| hsa-let-7e-5p | 1 | 1.87112 | 0.0000284 |
| hsa-miR-15a-5p | 1 | 1.82611 | 0.000139 |
| hsa-miR-34b-5p | 1 | 2.96466 | 0.00000549 |
| hsa-miR-181a-2-3p | 1 | 3.66278 | 0.00000373 |
| hsa-miR-382-5p | 1 | 1.92496 | 0.00183 |
| hsa-miR-181a-3p | 1 | 3.99562 | 0.0000962 |
| hsa-miR-450a-5p | 1 | 2.22079 | 0.000762 |
| hsa-miR-181a-5p | 1 | 2.23624 | 0.00000832 |
| hsa-miR-4734 | 1 | 1.92496 | 0.0083 |
| hsa-miR-181b-5p | 1 | 2.36646 | 0.00000455 |
| hsa-miR-503-5p | 1 | 2.82405 | 0.000113 |
| hsa-miR-181d-5p | 1 | 1.77575 | 0.000189 |
| hsa-miR-6516-3p | 1 | 1.8748 | 0.0185 |
| hsa-miR-182-5p | 1 | 3.41221 | 0.0000974 |
| hsa-miR-6865-3p | 1 | 1.92496 | 0.011 |
| hsa-miR-191-5p | 1 | 1.92496 | 0.00183 |
| hsa-miR-96-5p | 1 | 1.7984 | 0.000694 |
| hsa-miR-195-3p | 1 | 1.92204 | 0.0184 |
| hsa-miR-340-3p | 1 | 3.91811 | 0.000000995 |
| hsa-miR-200a-5p | 1 | 2.09222 | 0.0123 |
| hsa-miR-34a-5p | 1 | 3.25209 | 0.000001 |
| hsa-miR-205-3p | 1 | 1.92496 | 0.00183 |
| hsa-miR-34c-5p | 1 | 1.9939 | 0.0107 |
| hsa-miR-212-3p | 1 | 2.82634 | 0.00151 |
| hsa-miR-375 | 1 | 5.96267 | 0.0000154 |
| hsa-miR-21-3p | 1 | 2.08952 | 0.000766 |
| hsa-miR-409-3p | 1 | 2.83771 | 0.000457 |
| hsa-miR-21-5p | 1 | 3.36961 | 0.00000289 |
| hsa-miR-425-3p | 1 | 1.89186 | 0.0212 |
| hsa-miR-101-5p | 1 | 1.92496 | 0.00183 |
| hsa-miR-4652-3p | 1 | 1.92496 | 0.00809 |
| hsa-miR-10a-3p | 1 | 1.92496 | 0.011 |
| hsa-miR-4714-3p | 1 | 1.94417 | 0.0114 |
| hsa-miR-125a-5p | 1 | 1.61701 | 0.000208 |
| hsa-miR-483-3p | 1 | 2.58934 | 0.00132 |
| hsa-miR-542-3p | 1 | 1.89484 | 0.000694 |
| hsa-miR-495-3p | 1 | 1.93453 | 0.00187 |
| hsa-miR-564 | 1 | 1.53836 | 0.0115 |
| hsa-miR-132-3p | 1 | 1.67656 | 0.00289 |
| hsa-miR-579-3p | 1 | 2.51635 | 0.0246 |
| hsa-miR-222-5p | 1 | 1.92496 | 0.00374 |
| hsa-miR-5701 | 1 | 1.92496 | 0.011 |
| hsa-miR-23b-3p | 1 | 1.64119 | 0.000219 |
| hsa-miR-585-3p | 1 | 1.92496 | 0.00183 |
| hsa-miR-24-3p | 1 | 1.50572 | 0.000328 |
| hsa-miR-628-5p | 1 | 1.8349 | 0.0183 |
| hsa-miR-27b-3p | 1 | 1.6571 | 0.000151 |
| hsa-miR-6785-3p | 1 | 1.92496 | 0.0324 |
| hsa-miR-29b-1-5p | 1 | 1.72207 | 0.000416 |
| hsa-miR-6861-3p | 1 | 2.5377 | 0.00267 |
| hsa-miR-29b-3p | 1 | 1.72213 | 0.000182 |
| hsa-miR-6870-3p | 1 | 1.91645 | 0.0202 |
| hsa-miR-3065-5p | 1 | 2.67102 | 0.00033 |
| hsa-miR-744-5p | 1 | 1.77124 | 0.00998 |
| hsa-miR-31-3p | 1 | 5.3993 | 0.000000187 |
| hsa-miR-99b-3p | 1 | 1.92496 | 0.00809 |
| hsa-miR-31-5p | 1 | 4.45459 | 1.76E-08 |
| hsa-miR-32-5p | 1 | 2.61934 | 0.00453 |
| **Grand Total** | **85** | **2.660403412** | **0.003901135** |

**Supplementary Table 2** Predicted target genes of miRNA candidates.

| **miR-146B-5P** | **miR-221-3P** | **miR-222-3P** |
| --- | --- | --- |
| PDGFRA | MMP2 | HNRNPH1 |
| KIT | RBM6 | SPTBN2 |
| KCTD15 | TUB | SLFN11 |
| SFRP1 | DIRAS3 | MINK1 |
| S100A12 | MEOX2 | DIRAS3 |
| POMT2 | CXCL12 | RANBP10 |
|  | TIAM1 | DOCK5 |
|  | KIT | MROH1 |
|  | ANXA1 | KIT |
|  | DDX3Y | PRICKLE4 |
|  | TLE4 | EPB41L2 |
|  | CREBZF | PAN2 |
|  | KLF9 | SRRM2 |
|  | CENPT | ZEB2 |
|  | ZEB2 | KNSTRN |
|  | SPAG5 | MAT2A |
|  | NAIP |  |
|  | MAT2A |  |
